# Biopolymers as Food Packaging Materials

**DOI:** 10.3390/ijms21144942

**Published:** 2020-07-13

**Authors:** Raffaele Porta, Mohammed Sabbah, Prospero Di Pierro

**Affiliations:** 1 Department of Chemical Sciences, University of Naples “Federico II”, Montesantangelo Campus, via Cintia 4, 80126 Naples, Italy; dipierro@unina.it; 2 Department of Nutrition and Food Technology, An-Najah National University, P.O. Box 7 Nablus, Palestine; m.sabbah@najah.edu

**Keywords:** biopolymer, food packaging, bio-based and biodegradable materials, edible films

Oil-derived plastics are the most commonly used materials for packaging because of their features, low cost, and availability of resources for manufacturing [1]. Their annual production continues to increase and, currently, the packaging sector still accounts for over 40% of the total worldwide plastic consumption, mainly because of the wide range of properties of plastics, which can be tailored according to the product requirement [2]. Polyethylene, polyethylene terephthalate, polypropylene, polyvinyl chloride, and polystyrene are the most common packaging plastics and, being non-biodegradable, they are responsible for a huge amount of environmental pollution [1]. Consequently, the development, characterization, and use of bio-based and biodegradable polymers are increasingly gaining importance and multidisciplinary attention, since, following their disintegration and composting, the “bio-plastic” materials can be used as fertilizers and soil conditioners [3]. These environmentally friendly biopolymers can be either chemically synthesized from bio-derived monomers or directly extracted from biomass or industrial wastes. However, the biopolymer-derived materials often lack the performance features of conventional plastics, such as strength, flexibility, and barrier properties, even though numerous efforts are made to improve them by using different approaches. In fact, the storage and transport of any product, especially food, requires that the packaging is resistant and able to withstand any external conditions that the storage or transportation imposes. Moreover, packaging should preserve food quality, prolonging the product shelf life. Finally, the promotion of sales requires that the packaging appearance is able to attract customer attention, as well as that the packaging material could be printable to give all the needed information on the product ingredients. 

A high number of cutting-edge studies on biopolymers potentially usable in the areas of both food and non-food packaging have been carried out in the last decade [4]. Among these, different kinds of blends with a variety of additives, nanostructured materials, macro, micro, and nano bio-composites, and several waste-derived renewable materials have been produced.

The present Special Issue had its origin in the aforementioned context and has the objective to emphasize interdisciplinary research on the structure, morphology, processing, and properties, as well as possible applications, of innovative biopolymers to be used in food packaging. It is a collection of recent investigations detailing physical, mechanical, and barrier properties of different biopolymer-based films and coatings to bring to the attention of materials scientists, biochemists, and food technologists and biotechnologists. In particular, the Special Issue contains one review and nine experimental studies. The review provides an overview of different biopolymer-based materials containing polysaccharide and protein matrices reinforced with silver nanoparticles to prepare active packaging for food applications [5]. The physical and mechanical properties of the described edible films were improved by the presence of the silver nanoparticles and the authors suggested that they may be used as packaging materials for different types of food to even protect them against the growth of microorganisms. However, further research is absolutely needed to determine the optimum levels of silver nanoparticles that can be safely applied in the obtained nano-materials.

Conversely, the other nine articles described the effects of various additives used to improve the physicochemical and biological properties of different biodegradable materials, including studies related to nanotechnology and bio-nanocomposites for packaging applications

Potrc et al. [6] and Zemljič et al. [7] reported the great potential of polyethylene and polypropylene packaging foils, coated by chitosan and polyphenol colloidal formulations, as active (antioxidant and antimicrobial) packaging in the food industry. 

Tyuftin et al. [8] evaluated the physical properties of laminated edible films manufactured using gelatin, whey proteins, or sodium alginate, optimized through the addition of corn oil. Among these, the gelatin/alginate laminate containing corn oil exhibited the best performance.

Fang et al. [9] produced, by casting method and sonication, an effective flaxseed gum film incorporated with carvacrol-possessing antioxidant and antimicrobial properties.

Latos-Brozio et al. [10] obtained environmentally friendly materials based on the biodegradable aliphatic polyesters (polylactide and polyhydroxyalkanoate) enriched with plant functional additives (catechin and polydatin).

Diaz et al. [11] outlined the relevance of the use of flours as materials for biodegradable film preparation, due to the low cost as well as to the fact that flours are natural mixtures of compatible macromolecules. In particular, the authors reported that chickpea (*Cicer arietinum* L.) flour shows a very promising composition for the development of edible films. 

Feng et al. [12] described antioxidant edible coatings, made with whey protein nanofibrils and titanium dioxide nanotubes, able to limit microbial growth, reduce weight loss, and extend the shelf life of chilled beef. 

Sabbah et al. [13] focused on the possibility to obtain protein-based films by recovery and formulation procedures from seed oil cakes, potentially useful, especially as mulching sheets. In particular, films prepared from defatted cake waste obtained from *Nigella sativa* (black cumin) are described. 

Montava-Jordà et al. [14] highlighted the valorization of cotton waste from the textile industry for the development of sustainable and cost-competitive biopolymer composites. They observed that the incorporation of recycled cotton waste increased the hardness of bio-polyethylene terephtalate and, thus, the resultant biopolymer composite could be of interest in rigid food packaging and related applications.

In conclusion, the editors have made a conscious effort to select the articles of authors from various parts of the world representing diverse disciplines, including material science, biochemistry, biotechnology, microbial sciences, food technology, and packaging engineering, convinced that only a multi- and inter-disciplinary approach may contribute to the production, characterization, and application of environmentally friendly innovative materials capable of replacing, at least partially, the conventional plastics so far used in food packaging.
